# Automated Analysis Pipeline for Extracting Saccade, Pupil, and Blink Parameters Using Video-Based Eye Tracking

**DOI:** 10.3390/vision8010014

**Published:** 2024-03-18

**Authors:** Brian C. Coe, Jeff Huang, Donald C. Brien, Brian J. White, Rachel Yep, Douglas P. Munoz

**Affiliations:** 1 Centre for Neuroscience Studies, Queen’s University, Kingston, ON K7L 3N6, Canada; 1jh19@queensu.ca (J.H.); briend@queensu.ca (D.C.B.); brian.white@queensu.ca (B.J.W.); doug.munoz@queensu.ca (D.P.M.); 2 Sunnybrook Research Institute, Toronto, ON M4N 3M5, Canada; rachel.yep@sri.utoronto.ca

**Keywords:** saccade, pupil, blink, video-based eye tracking, automated saccade classification, automated blink classification, automated trial marking, post-saccadic slosh, post saccadic oscillations, pupil size, blincades, boomerang saccades

## Abstract

The tremendous increase in the use of video-based eye tracking has made it possible to collect eye tracking data from thousands of participants. The traditional procedures for the manual detection and classification of saccades and for trial categorization (e.g., correct vs. incorrect) are not viable for the large datasets being collected. Additionally, video-based eye trackers allow for the analysis of pupil responses and blink behaviors. Here, we present a detailed description of our pipeline for collecting, storing, and cleaning data, as well as for organizing participant codes, which are fairly lab-specific but nonetheless, are important precursory steps in establishing standardized pipelines. More importantly, we also include descriptions of the automated detection and classification of saccades, blinks, “blincades” (blinks occurring during saccades), and boomerang saccades (two nearly simultaneous saccades in opposite directions where speed-based algorithms fail to split them), This is almost entirely task-agnostic and can be used on a wide variety of data. We additionally describe novel findings regarding post-saccadic oscillations and provide a method to achieve more accurate estimates for saccade end points. Lastly, we describe the automated behavior classification for the interleaved pro/anti-saccade task (IPAST), a task that probes voluntary and inhibitory control. This pipeline was evaluated using data collected from 592 human participants between 5 and 93 years of age, making it robust enough to handle large clinical patient datasets. In summary, this pipeline has been optimized to consistently handle large datasets obtained from diverse study cohorts (i.e., developmental, aging, clinical) and collected across multiple laboratory sites.

## 1. Introduction

Eye tracking has a long and rich history of use as a tool to study behavior and brain function [1,2,3,4], including applications in basic science research, as well as in clinical studies. Video-based eye tracking technology has advanced to a state that not only incorporates high frequency sampling and high spatial fidelity, but which is also minimally invasive [5]. This has led to the opportunity to collect and combine large datasets from multiple laboratory sites [6]. To fully realize the potential of these large datasets, new tools are required for the automation and standardization of data analysis and reporting. These tools need to be centralized so that all data is analyzed in the same manner. By using a singular and standardized pipeline, the well-known variability of user-based, hands-on detection and classification of events, like saccade onset, is replaced with an objective and reliable analysis, combined with a trial marking system that is also reliable and repeatable.

Video-based eye tracking uses the pupil to estimate gaze position, and the pupil is defined by the ever-changing iris. This can detrimentally affect the spatial accuracy of video-based eye tracking because the orientation of the iris relative to the rest of the eye changes, creating post saccadic oscillations (PSO), which have been attributed to several causes [7,8]. One such explanation is due to fluid dynamics and rotational acceleration that cause the iris to continue to move after the eye itself has stopped (overshooting), creating an inaccurate prolonged saccade that is then followed by a rebound. When these overshoots and rebounds become extreme, it is clinically known as iridodonesis [9]. Here we show that this “tremulousness” is also present in some healthy control subjects, albeit at a much smaller scale and with highly variable incidence. These PSOs cause inaccuracy in achieving an accurate estimate of saccade end-point. We describe a novel method to improve saccade endpoint accuracy by incorporating these PSOs into the actual saccades. As many of the methods for the studies from our lab have been honed and repeated for numerous publications, it seems appropriate to provide a singular description for the techniques that have been developed over time and which we currently employ.

The goal of this methods paper is to describe an automated analysis pipeline that we have developed and implemented in numerous studies in which we used 500 Hz video-based eye tracking to collect data from healthy participants across the human life span, as well as from neurological and psychiatric clinical populations. We describe the details for large scale data collection, as well as the extraction of key saccade, pupil, and blink parameters. We are now employing this automated pipeline in numerous control and clinical studies [10,11,12,13,14]. It is not an aim of this paper to contrast our methods with those of other studies that may focus on only one component of eye-tracking or one clinical or control test group. Rather, the aim is to describe our automated pipeline created for the analysis of multiple datasets collected from different sites. Here, we only present data from healthy controls to describe the analysis pipeline that automates and optimizes data analysis from video-based eye tracking. In this way, we can extract precisely the same measures of eye position, pupil size, blink probability, and task performance from hundreds of participants from numerous data collection sites in a matter of minutes.

## 2. Methods

### 2.1. Equipment

During the eye tracking procedures, the participants were seated in a dark room, with their heads positioned comfortably on a head rest. The participants were seated so that their eyes were 60 cm away from a 17-inch LCD monitor (1280 × 1024 pixels, 32-bit color, 60 Hz refresh rate). An infrared video-based eye tracker (EyeLink 1000 Plus, SR Research Ltd., Ottawa, ON, Canada) was used to track monocular eye position at a sampling rate of 500 Hz. We found that higher sampling rates only added to the computational effort and doubled file sizes, but did not contribute to SRT accuracy. Different recording rates, screen sizes, and distances can be used, but they must be encoded into the output file for proper automation. The calibration and validation procedures were performed for each participant prior to the start of each task to ensure appropriate eye tracking accuracy. When possible, the 9-point array was used. However, in situations where calibration was difficult, a 5-point array was used.

### 2.2. Tasks

**Figure 1:**
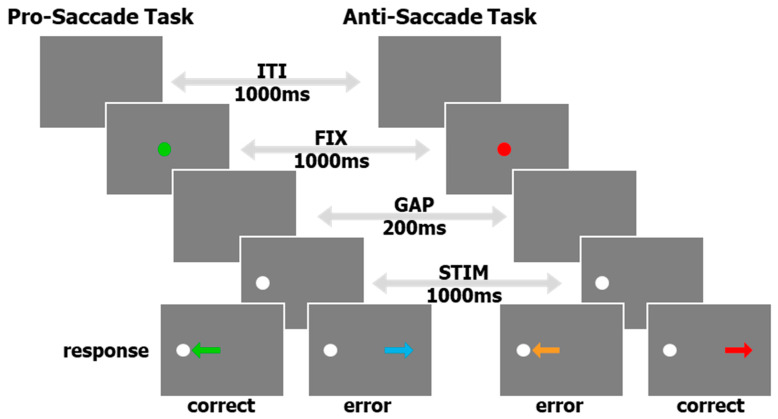
A schematic of the timing and visual aspects of the IPAST. Arrows appearing in the ‘response’ row indicate saccade direction, and are color coded by the result of the trial.

Although most of the collection, curation, and pre-processing described below can be implemented for multiple eye tracking tasks, here, we concentrate on data from an interleaved pro/anti-saccade task (IPAST, see Figure 1) and some free-viewing of common scenes (FreeViewing) originally created to measure saliency and attention deficits without employing a structured task. The IPAST data is structured and provides specific behavioral metrics, like reaction time and error rate, whereas the FreeViewing data is unstructured and is conducive to recording a more robust variety of natural saccades. In general, the results of these two tasks were collected on the same day. Each IPAST trial began with 1000 ms of an inter-trial interval (ITI) consisting of a blank black background (0.1 cd/m^2^). Next, a central fixation point (FIX; 0.5° diameter dot, 44 cd/m^2^) was displayed for 1000 ms. The color of the FIX conveyed the instruction for each trial (green: pro-saccade trial, PRO; red: anti-saccade trial, ANTI). The FIX was then removed, and the screen was blank again for 200 ms (gap period, GAP). Finally, a peripheral stimulus (STIM; 0.5° diameter dot; gray, 62 cd/m^2^) appeared 10° horizontally to the left or right of the FIX location. Each trial lasted 3200 ms. For the PRO trials, the participants were instructed to perform a saccade to the STIM as soon as it appeared (green arrow, bottom of Figure 1). In the ANTI trials, the participants were instructed to look toward the direction opposite of the STIM (red arrow, bottom of Figure 1). Trials in which the first saccade after the STIM onset was in the direction opposite of the trial instruction are known as Direction Errors (cyan and orange arrows, bottom of Figure 1). The task conditions (PRO or ANTI), as well as STIM locations (left or right), were pseudo-randomly interleaved with equal frequency. The IPAST experiment consisted of two blocks, with a short break in between, of 120 trials, each lasting approximately 20 min in total. Special codes were logged in the eye tracker’s raw data file (*.EDF) to represent the timing of the events described above and the nature of each trial, as well as recording resolution. We also included screen metrics, such as screen size, pixel resolution, and distance between the participant and the screen, in the raw *.EDF file. The timing of all stimuli was verified by an external photosensor at each installation site prior to data recording.

For FreeViewing data collection, the videos were displayed on the same monitor as the IPAST using custom software in Ubuntu 13 that interfaced with the eye tracker via the SR Research API. All participants viewed a total of 10 movies at 30 fps and with no audio. Each movie was contained in an independent file, was approximately 1 min in duration, and consisted of 15~17 video clips that were 2~5 s each in duration. The video clips featured scenes that may contain humans, animals, buildings, cars, and/or natural scenes. The clips in each movie instantaneously changed from one clip to the next and were presented in a fixed sequence within each movie. The order of the first five movies and then the second five movies was randomized between participants. The task required no instructions, and the participants simply viewed the movies. The timing of video onset was verified by an external photosensor.

### 2.3. Participants

All experimental procedures have been assessed and approved by the Queen’s University Health Sciences and Affiliated Teaching Hospitals Research Ethics Board. Similar to the study by Yep et al. [11], individuals between the ages of 5–93 were recruited from the greater Kingston, Ontario, area using online and newspaper announcements. The participants reported no history of neurological or psychiatric illness and had normal or corrected-to-normal vision. A signed informed consent form was obtained from all individuals over the age of 18. An assent form, in addition to a parent’s or guardian’s informed consent form, was obtained from all individuals under the age of 18. Study sessions lasted approximately one hour. For their participation, participants were remunerated USD 20. 

**Figure 2:**
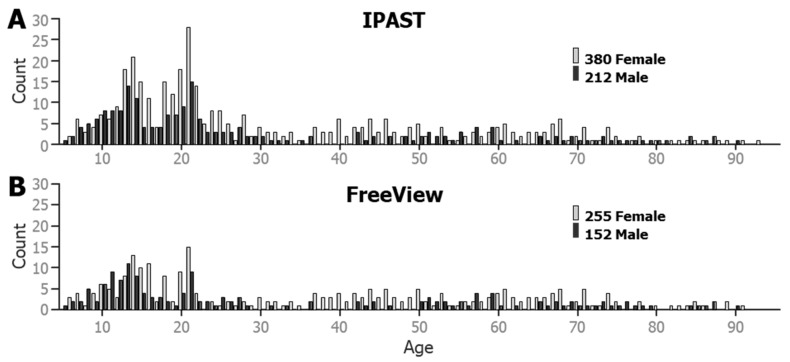
Age-based histograms of the participant database, by gender. (**A**) Age and gender of the participants that performed the IPAST. (**B**) Age and gender of the participants that participated in Free Viewing.

In total, 592 participants across a dynamic age range contributed to the current dataset (see Figure 2). All participants performed at least one block of the IPAST and provided at least 30 viable saccade trials from both the PRO and the ANTI tasks. Viable saccades are described in the Section 2.4 below. A total of 407 participants also completed the FreeViewing task.

### 2.4. Collection, Curation, and Pre-Processing

All raw eye tracking data files (*.EDF) were stored in task-specific folders (i.e., each run/block had its own folder) inside of one participant folder, which was labeled using a 4-digit participant identifier and the date of recording (yyyymmdd, in descending order for easier sorting). An online database of available participant identifiers was set up so that numerous data collection sites could work in unison to assure that each participant was assigned a unique identifier.

### 2.5. Collection

Basic demographic information about each participant was digitally collected immediately prior to each of the two IPAST runs/blocks via experimenter input to a custom front-end program written in Java. This program performed error checking on our naming conventions, launched the IPAST automatically, and then saved the eye tracking data (*.EDF file) and the demographic information (*.txt file) in the previously described task-specific folder to reduce errors in data collection and participant identification. FreeViewing data collection took place following the IPAST task and was stored in its run-specific folder; all three run-specific folders (2 IPAST and 1 FreeViewing) were stored inside one participant folder.

### 2.6. File Conversion

All data processing was completed using custom software in MATLAB R2020a~2022a (MathWorks, www.mathworks.com). Raw *.EDF files were converted to MATLAB files (*_EDF.MAT) via custom code using EyeLink API (SR Research Ltd., Ottawa, ON, Canada). This was conducted in a task-agnostic fashion; thus, the process can be used on any EDF file recorded by our lab. There were minimal changes to the data during this conversion. The first timestamp in each file was stored separately and then subtracted from all other timestamps in the file. This allowed for the data size to be reduced to a “single” (32-bit precision) instead of a “double” size (64-bit precision). Other scores were similarly reduced in size, while still maintaining the full range of values. These small changes decreased the converted data size by approximately 25% of the original data size and drastically decreased the time it took to transfer, load, and analyze the files. Timestamps, horizontal and vertical eye position, and pupil area data (T, X, Y, A, respectively) were collected at 500 Hz. All of our projects are created with a few standard tags that not all EyeLink 1000 users may employ. All task events (e.g., trial start, FIX onset, STIM onset, etc.) were recorded with a corresponding timestamp in the *.EDF file. All event timestamps were rounded to match the recording rate (i.e., multiples of 2 for 500 Hz) for easier alignment with the eye data. Raw X, Y, and A data were recorded in pixels. The converted data file (*_EDF.mat) was then saved in the original raw data folder for quick and easy loading.

### 2.7. Cleaning and Combining

The EyeLink 1000 pauses recording in between trials, so the data is not continuous throughout the entire recording; thus, further analysis was performed on a trial-by-trial basis. The next step was to load the converted *_EDF.mat file using custom task-specific functions that would first separate the T, X, Y, and A data into individual trials, using common event stamps implemented by the EyeLink Experiment Builder task creation software. Although extremely rare, timestamp gaps or repeats were fixed if they occurred; either by linear interpolation at 2 ms for the missing timestamps, and the corresponding X, Y, and A, or by removing the duplicate timestamps and the corresponding values, respectively. The converted data file (*_EDF.mat) for each IPAST block was combined with its paired metadata file (*.txt), created by the front-end Java program. For each FreeViewing block, we used a metadata file from an accompanying IPAST block in the same participant folder, as they were run on the same day. The pixel-based X and Y data were transformed into degrees of visual angle using screen size and the distance from the participant to the screen, digitally added to the raw *.EDF file. The EyeLink 1000 has the ability, known as a “drift-correct”, to update the initial calibration at regular intervals between trials during the experiment. For each of the intervals between “drift-corrections”, we determined the mode of eye position during a known fixation period for each trial to best assess the actual fixation of the participants, as well as noise in the eye data [15]. This was used to create a post-hoc X and Y adjustment to better estimate the actual fixation of each participant and to determine an adjustable threshold for saccade detection (described in detail below). These adjustments could also be used as a measure of eye tracker accuracy that would complement other similar measures [16]. This new data structure was then entered into an automated pipeline that initially performs pre-processing (described below) and then conducts task-specific trial-by-trial auto-marking for task performance (Figure 1, bottom row) and behavioral markers (described in Section 2.8).

**Figure 3:**
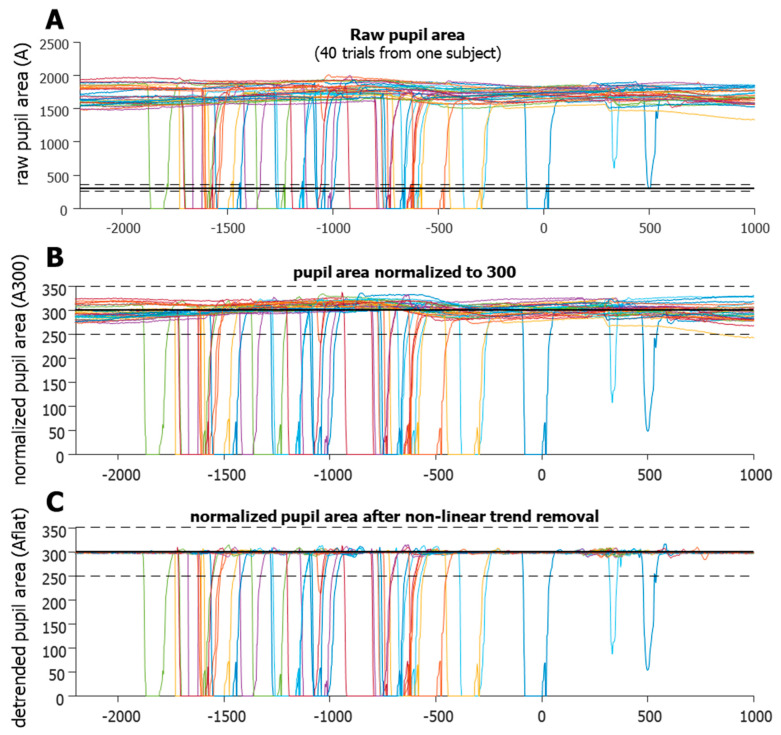
Blink detection algorithm. (**A**). Raw data (in pixels) from 40 trials of one participant. (**B**). Normalized data (trial by trial) forced to a mean of 300 (now in arbitrary units). Notice that in some cases, the natural fluctuations make using a fixed threshold (dashed horizontal line) for blink onset untrustworthy. (**C**). The benefits of non-linear trend removal, making the use of a fixed threshold for blink detection more robust. It can now detect partial blinks, yet not be triggered by large fluctuations, as these were removed. Colors simply indicate separate trials.

Smoothing is a common method used to help clarify high frequency data. We chose to use MATLAB’s *filtfilt* function that “performs zero-phase digital filtering by processing the input data, x, in both the forward and reverse directions” (MATLAB help documentation). For a review of different smoothing applications at different recording rates, see Mack et al. [17]. We used box-shaped transfer function coefficients for the *filtfilt* function, which is similar to a moving average with a given width. Thus, the larger the width, the larger the window, and the greater the smoothing. This method was used for all filtering, as this technique was the simplest form, it treated all data points equally (saccades or otherwise), it made the fewest assumptions, and it exerted the smallest effect on the starts and ends of changes in eye velocities (see Mack et al. [17], Figure 3). The MATLAB code was made dynamic by adjusting a single variable: the width.

(1) s_X=filtfilt(ones(1,w),w,X);

where *w* is the width of the window for smoothing, *X* is the data to be smoothed, and the “ones” function gives equal weight to all data points in the window.

In a similar manner, differential calculations for instantaneous *X* and *Y* velocities were also calculated in a zero-phase manner. To calculate an instantaneous differential for a given point in time, the previous data point was subtracted from the following data point and then divided by 2. For the first and last data points, a simple 2-point difference was used.

(2) $$vel_{X}=\frac{\left[X_{2}-X_{1},\frac{X_{3:n}-X_{1:n-2}}{2},X_{n}-X_{n-1}\right]}{\Delta s}$$

where *X* is the vector of horizontal position (replace with *Y* for the vertical position), *n* is the number of data points, and Δ*s* is the difference in time in seconds between each datapoint (Δ*s* = 0.002 for 500 Hz recording). Eye speed in degrees per second was determined via the usual Euclidean process, using both of the smoothed eye velocity vectors.

### 2.8. Pre-Processing

Once the data were separated by trials using custom task-specific functions, the data were run through the following two pre-processing steps, which are task-agnostic.

#### 2.8.1. Blink Detection and Definition

While video-based eye tracking is by far the easiest and least invasive form of eye tracking, it suffers from data loss during several situations. Blinks can cause a full, or even partial, occlusion of the pupil, which results in data loss. At first deemed a nuisance, these moments of data loss have recently become useful for studying a vast variety of blink behaviors [18,19,20,21]. This data loss during eye tracking can occur for a number of different reasons, and is not caused only by blinks. For example, eye makeup like mascara can cause the pupil detection algorithm to include the lashes into the pupil estimate, creating an unstable and noisy signal. Thus, the most important issue is not to merely detect this loss, but to classify it properly as either a true blink or some other loss.

In order to better isolate when data lost was due specifically to blinks, several steps were taken to clarify the pupil area data. As the pupil area data (*A*) can be quite variable (Figure 3A) the first step was to normalize each trial to have the same average area.

(3) $$A300=\frac{A}{mean\left(A\left(A>10\right)\right)}*300$$

The raw pupil area values were the number of pixels of the eye’s image in the tracking camera, and generally ranged from hundreds to thousands. Thus, values below 10 were not included in the calculation of the mean, and the number 300 was arbitrarily chosen. This determined that the non-zero mean of the pupil area was fixed to 300 for each trial (*A*300, Figure 3B). The next step was to model the low-frequency modulation of *A*300 over time so that it could be removed to create a flattened *A*300. The goal of this step was to help clarify the sections of data that were indicative of eye loss. We smoothed pupil velocity (i.e., a change in *A*300) using a 3-point kernel, and this smoothed velocity (sVEL) was used to flag high velocity data for removal and replacement. A model of the low-frequency modulation was created using a copy of the A300 data, in which the high velocity (sVEL > 1000) and abnormal area (*A*300 < 200 or *A*300 > 400) data were replaced using linear interpolation. All data between the data point that preceded the section and data point that followed the section were interpolated. This model was then smoothed using a large 50-point kernel to help remove the high-frequency modulation of the area signal. This model was subtracted from the *A*300 to remove low-frequency, non-linear trends to create a flattened profile that exaggerated high velocity changes in area (*A*flat, Figure 3C), which greatly aided in the detection of the start and end points of data loss. These were defined as *A*flat < 250 or *A*flat > 350 (black dashed lines in Figure 2). We noticed that data loss was preceded and followed by drastic fluctuations in pupil area data, indicating that an actual blink lasted longer than the loss of data. Typically, the A300 would drop off quickly just prior to data loss. This was presumably due to the eyelid progressively occluding the pupil, thus reducing the number of pixels delineated by the eye tracker as the pupil. Likewise, after the data loss, the *A*300 would recover from near zero and waver around 300, as more of the pupil became visible to the camera again. To better define the actual duration of a blink, these fluctuations were considered part of the blink. Once start and end points of the data loss were detected, we used the smoothed absolute velocity (saVEL) of the *A*300 to determine the start and end of the full blink using a dynamic threshold per trial, similar to using speed for saccade detection.

**Figure 4:**
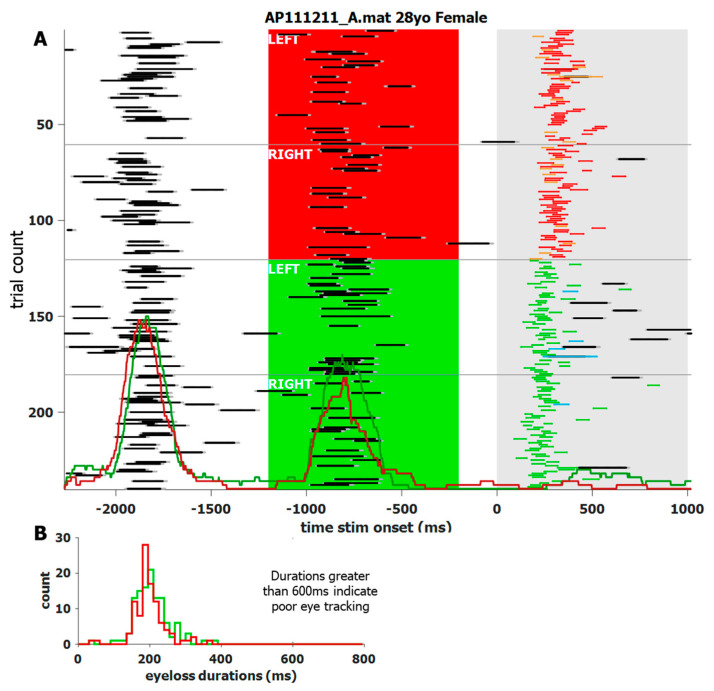
Screen shot of a custom application to visualize the blink behavior of a single participant with a high blink rate. (**A**) trial-by-trial recap of what transpired. The trials start with the ITI, followed by the colored FIX, and then the STIM onset at 0 ms. The trials are re-ordered by task (ANTI above PRO) and STIM location (left above right), and are indicated by white text. Data loss is indicated by black lines, bordered by dark grey, to indicate the full blink length. Task related saccades, between 90 and 800 ms, are color-coded to match Figure 1. Red and green histograms overlaid in (**A**) indicate probability, not just onset, of a blink for ANTI and PRO saccades, respectively. Their heights are proportional to the number of total trials. Red and green histograms overlaid in (**B**) indicate blink duration by task.

We noticed that blinks generally had stereotypical durations and patterns in the pupil area data. We also noticed that each individual’s blinks affect the data slightly differently. We used the duration of data loss to categorize whether a loss in data was due to a blink or due to some other interference. Although the blink detection analysis is task-agnostic, task specific software was used to visualize the output. For example, Figure 4 shows a screenshot of the custom software (MATLAB 2020a~2022a) used to visualize an individual’s blink behavior during the IPAST. The trials are reordered by task (ANTI over PRO) and by STIM location (left over right). For this subject, and most others, the ITI and FIX intervals show an increased number of blinks in an organized manner, despite no such instructions to participants. The main window also shows blink probability (not just onset) histograms overlaid at the bottom. The panel on the lower left shows the histograms of blink durations and are color coded by task. This individual was chosen for their consistent blink behavior, which was common among most participants. However, there were some individuals who rarely blinked at all.

#### 2.8.2. Saccade Detection and Definition

The detection of saccade onset was a straightforward process, determining a speed threshold and then finding the first data point that was above it, with consecutive data points remaining above this threshold for a given duration. To determine the dynamic threshold for each trial, the background noise during a fixation epoch, where eye speed was below a fixed threshold of 50°/s, was used. Thus, noisier trials were assigned a higher threshold to avoid numerous false positives. We used data below this fixed threshold to find the mean and standard deviation of the background noise. Our dynamic threshold for each trial was defined as the mean plus 2.5 times the standard deviation, but was never less than 20°/s. The eye speed had to remain above this dynamic threshold for 10 ms to be identified as a saccade onset. Lastly, two Z scores were calculated for each saccade, based on the main sequence [22], and could be used to validate each saccade. That procedure is described in detail in the Section 2.8.3 below. Recently, more complex algorithms have been published [23,24]; however, our main goal was to maintain consistency with previously published work.

The end point of the saccades, however, could be quite difficult to properly determine due to well-known aspects of video-based recording [7,25,26,27,28] and the ways in which they differ from the classical search coil technique [5,29,30].

**Figure 5:**
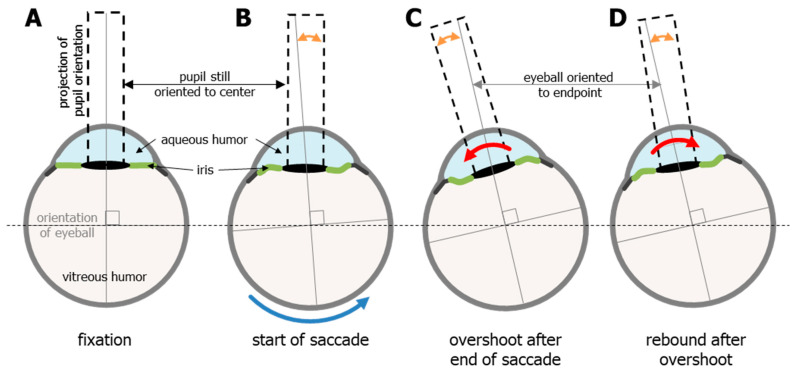
The effects of rotational acceleration and deceleration on the shape of the iris and the orientation of the pupil (dashed black rectangles indicate orientation) in relation to the rest of the eye. (**A**) the eye at rest, or fixation. The orange double-headed arrows in panels (**B**–**D**) show the discrepancy between the orientation of the eye (which indicates actual gaze) and the orientation of the pupil (used by video-based eye trackers to estimate gaze). (**B**). The effects of the inertia of the aqueous humor may cause a delay in the motion of the iris/pupil, although this is thought to be minimal. The last two stages are overshoot (**C**) and rebound (**D**), which alternate and diminish with each cycle until equilibrium (seen in fixation) is once again reached. This sloshing of the aqueous humor can cause or increase the well know post-saccadic oscillations.

Video-based eye tracking uses the pupil to estimate gaze, and the pupil is defined by the everchanging iris. The iris not only expands and contracts to change the diameter of the pupil, but the orientation of the iris relative to the rest of the eye also changes due to fluid dynamics and rotational acceleration. This means that the pupil/iris orientation does not always align with the orientation of the eye and gaze [7]. The forces on the iris have previously been described in detail [9,31]. To summarize briefly, the lens and iris structures are situated in between two independent fluid bodies: the aqueous humor in front (light blue, Figure 5), and the much larger vitreous humor (light grey, Figure 5) that fills most of the eye, and these two systems do not connect. The vitreous humor is a closed (jelly-like) system that makes up the center-of-mass for the eye, whereas the aqueous humor is a flowing system (more like cerebrospinal fluid) and is on the periphery of the center-of-mass [9,31].

This arrangement causes the flexible iris to create something that the field of fluid dynamics refers to as a fluid–structure interaction. The rotational acceleration and deceleration of the eye creates a slosh dynamics problem (https://en.wikipedia.org/wiki/Slosh_dynamics, accessed on 1 January 2020) between two bodies of fluid, with a flexible structure in between. A full and proper discussion of fluid dynamics is beyond the scope of this paper; however, the mere acknowledgement of these issues enables a better understanding of the mechanical characteristics of the iris when the eye moves. Because the iris is flexible, the rotational acceleration of the eye and the inertia of the aqueous humor induces pressure strain on the solid structures of the eye that lie between the two liquid parts of the eye. Upon reaching a fixed (or zero) speed, this pressure is oscillatory and diminishes with each cycle until equilibrium is once again reached (https://en.wikipedia.org/wiki/Fluid-structure_interaction, accessed on 1 January 2020). The clearest visualization of this oscillatory motion of the iris is seen in the condition known as iridodonesis (numerous videos of this condition can be found on YouTube). Importantly, even healthy eyes exhibit this oscillatory motion, albeit to a much lesser degree, and it interferes with the proper detection of the end of an eye movement. It should also be noted that this oscillatory motion can be caused, or increased, by the iris structures being pulled by the lens [8].

**Figure 6:**
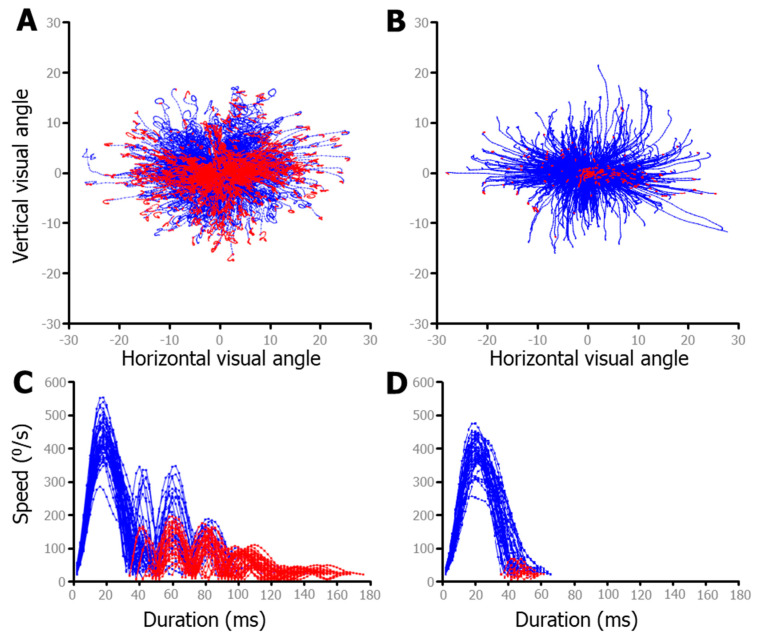
Fixed saccade mapping (FSM) above-speed plots of free-viewing data from two participants. Red data indicate the part of the saccade after the speed dropped below the threshold, but due to post saccadic slosh, motion continued. Some individuals exhibit much more mobility of the iris, leading to greater post-saccadic slosh. (**A**). The participant showing the most post-saccadic slosh. (**B**). The participant exhibiting the least post-saccadic slosh. (**C**,**D**) Speed plots for a sample of each individual’s (directly above) saccades. To ensure comparison of similar saccades, only rightward saccades made within ±22.5° of 0° and with amplitudes from 8 to 12° were plotted in (**C**,**D**).

To better illustrate the effects of the post-saccadic slosh, data from the FreeViewing task were used, as there was a larger variety of saccades during this unstructured data collection as compared to during the IPAST. Figure 6 shows saccade data from 2 individuals from our dataset; one with the most slosh detected (74yo female; Figure 6A) and the other with the least (46yo female; Figure 6B). The top two panels show a fixed saccade map (FSM), in which the starting point of each saccade was plotted from the center. The bottom two panels show speed traces of only rightward saccades of approximately 10°, in order to make cleaner comparisons. If only a fixed threshold was used to detect the end of the saccade, in the same way as detecting the start of the saccade, only the blue data points would have been considered as part of the main saccade. The following red data points represented data after the velocity passed below the threshold and would have been inappropriately classified as sperate micro saccades (e.g., saccades less than 2 degrees). The red data points indicate data that was added to the main saccade by detecting slosh-generated post-saccadic oscillations (PSO) that were supra-threshold (e.g., >20 °/s), close in time to the main saccade (<40 ms between), and smaller in amplitude (between 0.5 and 5°). Ignoring these oscillations generally led to inaccurate saccade end points. These were often hypermetric, as the eye would stop, but the iris would overshoot due to the fluid dynamics discussed above. For many people, including these PSO increases the accuracy of the saccadic end point, but it clearly overestimates the duration (Figure 6C,D). Of particular interest is the fact that the horizontal saccades had a predominantly horizontal wobble, whereas the oblique saccades oscillated in a circular pattern (see the end points in Figure 6A), which is indicative of independent vertical and horizontal slosh patterns.

#### 2.8.3. Saccade Data Tables

For each block of data collection, a table of saccade information was created. Each row represented one saccade, with columns indicating: trial number, start and end point, peak velocity, acceleration, amplitude, angle, and duration. Additional information was also included, and it is discussed below.

**Figure 7:**
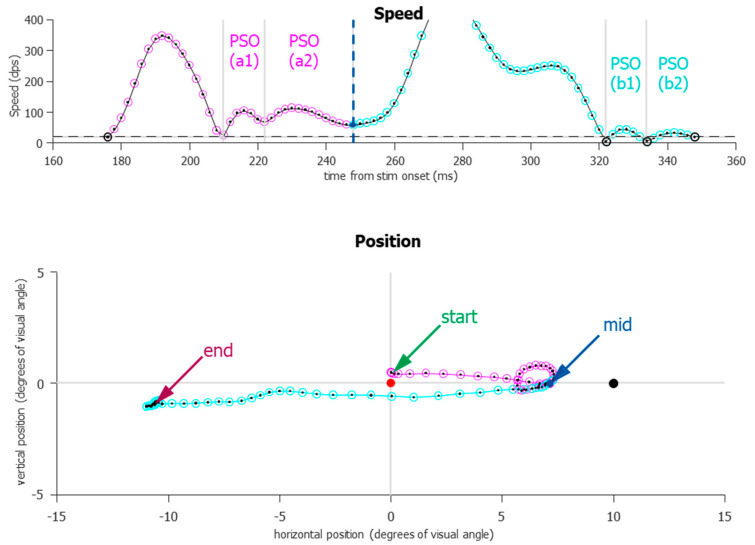
An example of a “boomerang” saccade that was successfully split. All data points in the figure were originally marked as part of one saccade. Saccade speed (dps) is plotted relative to STIM onset and is shown directly over the saccade path. Only four data points (highlighted in black) occurred below the speed threshold (dashed horizontal line, 20 dps), and two of them delineate PSOs (b1,b2), which are considered part of the saccade for better endpoint accuracy. Grey vertical lines indicate local nadirs. One local nadir (blue dot, labeled “mid”) was used to delineate the two saccades, which are color-coded magenta and cyan. In the position figure, the center red FIX indicates that this was an anti-saccade trial. The black dot on the right (10 degrees) indicates where the STIM was presented. Originally, this would have been marked as a correct anti-saccade that started at the FIX and ended near the correct location. After the split, there is a clear direction error toward the STIM, followed by a corrective saccade.

For the vast majority of saccades, the onset and offset (with the understanding of the post-saccadic slosh) were relatively easy to detect and define. However, two prominent exceptions still remained, one of them being more common in the IPAST. During an ANTI trial, participants occasionally exhibited what we call “boomerang” saccades (Figure 7). These are eye movements in which the initial trajectory and the final trajectory are diametrically opposed and can be indicative of the simultaneous planning of two saccades [32]. This occurred most frequently when a saccade was triggered to the error location but was immediately corrected and redirected toward the correct location, or sometimes returned to the fixation point and was followed by another saccade to the correct direction. What makes these boomerang saccades difficult to separate is that the velocity never drops below the speed threshold; thus, previous automated algorithms would define these two actions as one single saccade, which would start at the fixation and end at the correct location, or, in the case in which the boomerang saccade returned to fixation, as a micro saccade that starts and ends near fixation, followed by a correct saccade. This caused incorrect trials to be wrongly marked as correct trials (see Section 2.12). After the start and end of each saccade was detected, another step was used to detect whether the initial direction and final direction are opposite and align with the error and correct locations. Boomerang saccades should also show abnormal ratios for both duration and amplitude. This singular boomerang saccade was then split at a local nadir in the speed, even though it was above the threshold. Doing so revealed both error-to-correct boomerang saccades and correct-to-error boomerang saccades, the latter being extremely rare.

There were also incidents in which eye tracking was lost during an eye movement. This could be due to a blink or due to other eye tracking issues. These “blincades” were included in the table of saccades, with a tag indicating that some data was lost during the change in eye position.

In some situations, a normal blink can lead the eye tracker to falsely indicate an upward “blincade”, with data loss, followed by a downward “saccade”, as the tracker regains the pupil. This would lead to the mis-categorization of an actual blink (where no eye movement took place) as two saccades: a blincade upward immediately followed by a noisy saccade back down to the original position. For individuals who blink more frequently, this resulted in numerous fixation-break false-positives (see Section 2.11) and highly aberrant saccade metrics. In the situation where the automated process found an upward blincade, immediately followed by downward saccade and the end point was within 2° of the start point, these two events were combined and marked as a blink, not as two saccades. If the end and start point were farther than 2°apart, the two events were combined as a single blincade, with a linearly-interpolated trajectory from start to end. These blincades can be used for behavioral analysis, but not for saccade metrics.

As video-based eye tracking can be noisy, we know that some of the start points and end points of saccades can be misidentified; thus, a special process was conducted to indicate how consistent the saccades were for each participant. For each block of data, the main sequence (peak velocity vs. amplitude and peak velocity vs. duration) was calculated and modeled using a smoothing spline in MATLAB.

(4) fit(AMPL,pVEL,′smoothingspline′, ′SmoothingParam′,0.01);

Saccades with known issues, such as missing data, boomerang saccades, or blincades, were removed from the fitting process. Then, the Z-scores were calculated for each residual. This fit was used to calculate the main sequence Z-scores (MaSeZs) for each saccade and were added to the saccade table discussed above. These MaSeZs could be used to rank how “normal” a saccade was for that participant and whether its metrics should be used to calculate eye movement scores. For example, when calculating a participant’s mean peak velocity, only saccades with an MaSeZs less then 3.29 (*p* < 0.001) were used, as a Z-score greater than that is indicative of some eye tracking issue in which the behavior can be detected, but the instantaneous eye tracking was fouled.

### 2.9. IPAST Pipeline

The pre-processing steps described above could be performed on eye tracking data collected from most tasks, with minimal task-dependent alterations. The start and end of each trial must be identified, as well as the fixation epochs, to assist with the dynamic thresholds. We now describe the next steps in data analysis that are specific for the IPAST participants.

### 2.10. Pupillometry

In recent years, there has been an increased interest in pupil size, as it is readily available using video-based eye tracking [33,34]. Unlike the task-agnostic methods used for blink detection, for the IPAST blink analysis, the raw pupil area data, in pixel count, was used. Pupil size is a sensitive measure and can be affected by lighting conditions, blinks, noisy data, and eye position. To avoid the distortion of pupil measures due to movement and position, pupil size analysis was performed during the FIX and GAP periods for each trial, with several limitations. Only trials in which the eyes were stationary (no saccades > 2°), were looking in a central location (on or near the FIX) and did not exhibit too much data loss (<200 ms in duration) were used.

**Figure 8:**
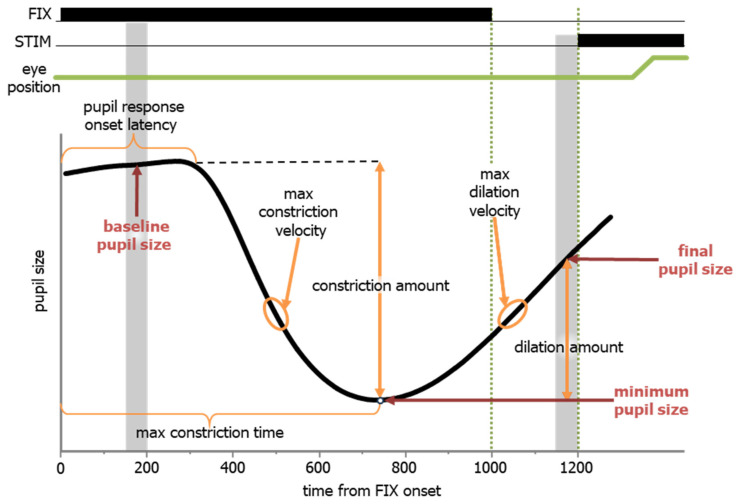
A schematic of the definitions for pupillometric measures during the IPAST.

The pupil has a characteristic response in the IPAST that differs between pro- and anti-saccade trials, and it reveals a cognitive component of top-down control. In response to the FIX onset, there was a stereotypical pupil response that began with constriction and was followed by dilation, prior to the predictable onset of the STIM. To capture the dynamics of the pupil response, we first calculated pupil size (based on area data) at three formative time points (Figure 8, red text). The baseline pupil size was calculated by averaging the pupil size during the epoch of 150 to 200 ms after FIX onset (Figure 8, first grey vertical bar), which is prior to any pupil response. The minimum pupil size was the nadir of the pupil size during the FIX epoch, and the time (relative to FIX onset) of the minimum pupil size was defined as the maximum constriction time. The final pupil size was calculated by averaging the pupil size during the epoch of 150 to 200 ms prior to STIM onset (Figure 8, second grey vertical bar, 1150 to 1200 ms relative to FIX). Additional calculations to help describe the dynamics of the pupil response were based on these values. They are indicated in the black text in Figure 8. The pupil response onset latency was defined as the earliest point at which pupil size significantly differed from the baseline pupil size, calculated using a 20 ms sliding window. The maximum constriction velocity was determined as the highest velocity between the pupil response onset latency and the maximum constriction time. The constriction amount was calculated as the difference between the baseline pupil size and the pupil size at maximum constriction. The maximum dilation velocity was determined as the highest velocity between the maximum constriction time and STIM onset. The dilation amount was calculated as the difference between the pupil size at maximum constriction and final pupil size.

### 2.11. IPAST Saccade Classification

**Figure 9:**
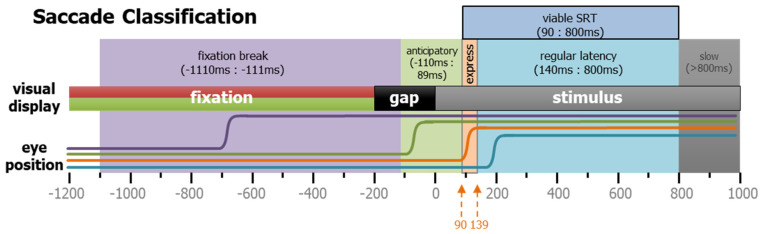
Task-related saccade classification. Saccades in the IPAST were categorized by timing, as well as direction. Here, four different types of rightward saccades are shown schematically. Purple indicates a fixation break, green shows an anticipatory saccade, orange represents an express saccade, and cyan reveals a regular latency saccade.

All detected saccades were classified according to when they occurred and their start and end positions. For viable saccades (see Table 1 and Figure 9), the saccadic reaction time (SRT) was defined as the time between the appearance of the STIM and the onset of the first subsequent saccade. Saccades towards the two potential stimulus locations that occurred during the GAP period were defined as anticipatory because they were equally likely to be either correct or direction errors, suggestive of a guessing behavior. We determined that the timing of the earliest possible visually triggered saccade was 90 ms after STIM appearance. Prior to this time in the PRO task, correct responses and direction errors were equally likely, but after this time, correct responses were triggered in excess of 95% of trials. The anticipatory window was set as −110 to 89 ms relative to STIM onset (light-green shaded area in Figure 9). The express and regular latency saccades have been defined and discussed previously [35,36,37]. Here, we delineate between the two types at 140 ms. Saccades that take place after 800 ms were extremely rare, but do occasionally occur. These extremely long SRT responses were used to classify the trial-type (see below), but as they represented the participants’ inattention, they were removed from the measurements of latency and saccade metrics.

### 2.12. IPAST Trial Classification

The final goal of this analysis was to classify each trial in the IPAST. Previously, trial classification was rather rudimentary, only including correct trials, incorrect trials, and “other” trials. These “other” trials were not used and could include trials lost due to poor data or some other behavior not specifically defined. Here, we were more thorough in defining each trial so that even the “other” trials could be useful.

Due to the larger variety of trial types, numerous behavioral metrics were calculated, producing standardized results. Table 1 contains the list of all possible trial types. Using the new categories of trial types, we could calculate not only error rates, but also non-compliance rates. For example, trials that fell under no-saccade, random saccade, and never fixated categories were summed up and used as a measure of non-compliance. Additionally, trials with fixation breaks and anticipatory saccades could also be independently measured. Trials in which fixation briefly lapsed, i.e., fixation was broken but was re-established before the FIX turned off, were allowed to be defined by the subsequent behavior. These lapses were marked in the saccade table and could be used for further investigations. Unmarked trials and data-lost trials were completely removed, and these constituted only 0.59% of attempted trials. This pipeline was able to read in data, classify blinks, categorize saccades, and automatically mark and categorize 99.96% of 137,896 IPAST trials from 592 participants in approximately 2 h (the data was recorded on a Linux mainframe server, and the code was run on a windows 10 PC with Intel® Core™ i7-4770 CPU @ 3.40 GHz).

We then defined the ANTI “error rate” as all direction error ANTI trials divided by the sum of all ANTI trials, including fixation break, anticipatory, and non-compliance trials, whereas “error ratio” was defined as all the direction error ANTI trials divided by the sum of only the correct and direction error ANTI trials. The “rate” calculation asks, “of all the ANTI trials where a measurable behavior took place, how many were direction errors?” The “ratio” calculation is more similar to the calculations of past methods, as it removed the fixation break, anticipatory, and non-compliance trials, and asked a slightly different question: “out of all the viable ANTI saccades (see Figure 8) made, how often did they fail to suppress the sensory driven signals?” For many participants, these values were similar. However, for those who struggled to understand the task and exhibited more fixation break, anticipatory, and/or non-compliant trials, the difference between these values was meaningful and warranted further investigation. Additionally, fixation break “rates”, anticipatory “rates”, and other similar scores were calculated using all trials.

## 3. Discussion

Here, we describe in detail our data analysis pipeline to automate and optimize data analysis from video-based eye tracking, in which we can extract measures of eye position, pupil size, and blinks. This process is not only faster than manual marking, but it is also far more consistent and produces repeatable measurements of behavior. These methods are not meant to be a standardized protocol for all anti-saccade research, but merely serve as a description of what we have done and why. Subsequently, this pipeline has been used to conduct a larger control database analysis on several measurements of behavior [11,14], as well as on clinical cohorts [10,12,13].

With an understanding of the nature of the PSOs, the accuracy of the spatial location of the saccade end-points has been improved. The described approach combined, post hoc, the PSOs with the main saccade when the speed dropped below the threshold between the two events. This was done for every saccade in a task-agnostic fashion. Similarly, every blink was also detected and categorized. Blinks were once a nuisance to overcome in measuring eye movement via video-based tracking, but they have recently become a behavioral marker in their own right [18,20]. Additionally, the trials that were once discarded can now be utilized for behavioral analysis. The fixation break, anticipatory, and the different non-compliance trials can be used as meaningful behavioral markers. Collectively, these steps can lead to the standardization of IPAST behavior across numerous data collection sites to create easily comparable scores.

### 3.1. Task Parameters

The “interleaved” and “gap” aspects of the IPAST were chosen for several reasons.

#### 3.1.1. Interleaved

Previous versions of the anti-saccade task [38,39] used a blocked design which isolated the pro-saccades from the anti-saccades in separate blocks of 100 or more consecutive trials. The block design is not ideal for contrasting pro- and anti-saccade behavior because the levels of concentration, arousal, and fatigue vary between blocks of different tasks and over time in a prolonged laboratory experiment. The interleaved version of the task requires more working memory [40], as the current trial’s rule must be updated and remembered upon STIM presentation on a trial-by-trial basis. Blocked and interleaved pro- and anti-saccade tasks have been compared directly [41], albeit using a rather different style of a pro- and anti-saccade task employing landmarks and other cues, which would affect saccade metrics. The study demonstrated a higher error rate in the interleaved sessions but no differences in regards to SRTs. We have also found that the dynamic aspect of the interleaved task also relieves monotony and increases the trial count for healthy participants. Lastly, it also assures that the visual, spatial, and temporal aspects of the two tasks (e.g., FIX and STIM luminance and timing) were as identical as possible.

#### 3.1.2. Gap

We also found that the “gap” paradigm (200 ms between FIX and STIM) improved the ability to measure the dynamic levels of inhibition needed to correctly perform the anti-saccade task, compared to the “step” (0 ms between FIX and STIM) or “overlap” (FIX stays on during STIM) paradigms. The presence of the gap period is known to lead to a reduction in the sustained activity of the fixation neurons in the superior colliculus [42], a reduction in reaction time, known as the gap effect [43], and more direction errors in the anti-saccade task [44]. Additionally, only two possible target locations were used, so there is a high degree of preparation, for both action and inhibition, during the task [45].

### 3.2. Post-Saccadic Oscillations

The types and sources of PSOs have been described for quite some time [7,8,23,25,26,31,46,47,48], and video-based eye tracking has been shown to record more PSOs than classical eye coil techniques [5,30,49]. Due to these PSOs, the end point of the saccades, as measured by video-based eye tracking, can be more difficult to detect [7,25,26]. Because PSOs affect the end points and duration of saccades, this has important implications for the accuracy of reported saccade metrics. Based on an understanding of what can happen to the eye during hard deceleration [9], we describe a method to obtain a more accurate measure of the actual end point of a saccade. We also described in detail how the eye-position data was smoothed because, similar to the discovery of Mack et al. [17] (see Figure 3), we have found that different smoothing techniques can actually increase the effects of the PSOs, further complicating saccade end point detection. Thus, when it comes to smoothing video-based eye tracking data, the simpler the method, the better.

The important idea to take away from this discussion is that to accurately measure the end position of a saccade, one must wait for the oscillations to end, with the caveat that this artificially elongates the duration of the saccades for individuals with greater slosh. In taking this approach, we sacrificed the accuracy of the duration measurement to achieve better accuracy regarding the end position. Future work should model the post-saccadic oscillations and form a better estimate of the timing of the end of the saccade. Also, similar to blinks, these PSOs could also be another potential behavioral marker.

### 3.3. Summary

In this paper, we describe our automated analysis features, which are important because they allow for the standardized quantification of saccades, blinks, and pupil response during any data recording, as well as IPAST performance across multiple cohorts of participants. To illustrate how important this is, this pipeline is currently being used to analyze large, variable datasets from diverse participant cohorts (i.e., developmental, aging, neurodegenerative, neuropsychiatric, and long-haul COVID), different study designs (i.e., baseline, longitudinal, and clinical intervention), and from collection sites all around the world (i.e., Canada, USA, Mexico, Brazil, Germany, and Ireland).

## Figures and Tables

**Table 1:** The trial categorization performance of the IPAST pipeline using data from 592 participants who attempted 137,896 trials.

Trial Type	Trial Description	Count	Percentage
Not marked	The trial has not been marked.	55	0.04%
Correct Pro-Saccade	A correct pro-saccade between 90 ms and 1000 ms after STIM appearance.	58,161	42.18%
Correct Anti-Saccade	A correct anti-saccade between 90 ms and 1000 ms after STIM appearance.	46,010	33.37%
Pro-Saccade Direction Error	An erroneous saccade to the anti-location during the pro-saccade trial between 90 ms and 1000 ms after STIM appearance.	1612	1.17%
Anti-Saccade Direction Error	An erroneous saccade to the STIM during the anti-saccade trial between 90 ms and 1000 ms after STIM appearance.	14,092	10.22%
Anticipatory Correct Pro-Saccade	A correct pro-saccade between −110 ms and 89 ms relative to STIM appearance.	2472	1.79%
Anticipatory Correct Anti-Saccade	A correct anti-saccade between −110 ms and 89 ms relative to STIM appearance.	1236	0.90%
Anticipatory Pro-Saccade Direction Error	An erroneous saccade to the anti-location during the pro-saccade trial between −110 ms and 89 ms relative to STIM appearance.	1914	1.39%
Anticipatory Anti-Saccade Direction Error	An erroneous saccade to the STIM during the anti-saccade trial between −110 ms and 89 ms relative to STIM appearance.	1538	1.12%
Fixation Break	The participant looked away from FIX prior to its disappearance and did not return prior to the end of the FIX epoch.	7848	5.69%
No Saccade	The participant fixated on the FIX but never exhibited a saccade after STIM appearance.	352	0.26%
Random Saccade	The participant fixated on the FIX and then made a saccade to a non-task relevant location after STIM appearance.	330	0.24%
Never Fixated	The participant never properly fixated on the FIX.	1521	1.10%
Eye Loss	Too much data was lost to properly analyze the result.	755	0.55%
**TOTAL**	The total from 592 participants (380 female).	137,896	100.00%

## Data Availability

Data is contained within the article.
